# Usability Evaluation of a Web-Based Support System for People With a Schizophrenia Diagnosis

**DOI:** 10.2196/jmir.1921

**Published:** 2012-02-06

**Authors:** Lian van der Krieke, Ando C Emerencia, Marco Aiello, Sjoerd Sytema

**Affiliations:** ^1^University Center for PsychiatryUniversity Medical Center GroningenUniversity of GroningenGroningenNetherlands; ^2^Johann Bernoulli Institute for Mathematics and Computer ScienceUniversity of GroningenGroningenNetherlands

**Keywords:** Schizophrenia, Web-Based systems, Recommendation systems, usability testing, self-management

## Abstract

**Background:**

Routine Outcome Monitoring (ROM) is a systematic way of assessing service users’ health conditions for the purpose of better aiding their care. ROM consists of various measures used to assess a service user’s physical, psychological, and social condition. While ROM is becoming increasingly important in the mental health care sector, one of its weaknesses is that ROM is not always sufficiently service user-oriented. First, clinicians tend to concentrate on those ROM results that provide information about clinical symptoms and functioning, whereas it has been suggested that a service user-oriented approach needs to focus on personal recovery. Second, service users have limited access to ROM results and they are often not equipped to interpret them. These problems need to be addressed, as access to resources and the opportunity to share decision making has been indicated as a prerequisite for service users to become a more equal partner in communication with their clinicians. Furthermore, shared decision making has been shown to improve the therapeutic alliance and to lead to better care.

**Objective:**

Our aim is to build a web-based support system which makes ROM results more accessible to service users and to provide them with more concrete and personalized information about their functioning (ie, symptoms, housing, social contacts) that they can use to discuss treatment options with their clinician. In this study, we will report on the usability of the web-based support system for service users with schizophrenia.

**Methods:**

First, we developed a prototype of a web-based support system in a multidisciplinary project team, including end-users. We then conducted a usability study of the support system consisting of (1) a heuristic evaluation, (2) a qualitative evaluation and (3) a quantitative evaluation.

**Results:**

Fifteen service users with a schizophrenia diagnosis and four information and communication technology (ICT) experts participated in the study. The results show that people with a schizophrenia diagnosis were able to use the support system easily. Furthermore, the content of the advice generated by the support system was considered meaningful and supportive.

**Conclusions:**

This study shows that the support system prototype has valuable potential to improve the ROM practice and it is worthwhile to further develop it into a more mature system. Furthermore, the results add to prior research into web applications for people with psychotic disorders, in that it shows that this group of end users can work with web-based and computer-based systems, despite the cognitive problems they experience.

## Introduction

Routine Outcome Monitoring (ROM) has become increasingly important in the mental health care sector. Although there is no universal definition, ROM can be described as the use of standard instruments to systematically and continuously assess aspects of mental health service users’ health for the purpose of better aiding their care [1]. The format of ROM varies between countries, but it usually consists of several quantitative measures used to assess a service user’s physical, psychological, and social condition. ROM is carried out for service users with a single diagnosis and short-term problems, as well as people with a severe mental illness. This latter group includes service users diagnosed with schizophrenia.

Schizophrenia is a mental disorder characterized by cognitive dysfunctions and abnormalities in perception of reality. People diagnosed with schizophrenia often experience hallucinations, delusions, and disorganized speech and thinking, accompanied by significant social and occupational problems [2]. Due to the complexity of this disorder and the diversity of care needed for service users diagnosed with schizophrenia, proper and frequent evaluation of treatment is particularly vital. That is why ROM offers much potential for better care of these people [3].

However, the effects of ROM on mental health care have been mixed. On the one hand, research shows that the use of outcome measures, combined with adequate feedback, helps clinicians to recognize and anticipate problems in individual treatment processes and to provide better care as a result [4-6]. On the other hand, ROM is not always used to in a way that empowers service users and improves shared decision making between service user and clinician [7,8]. One problem is that clinicians tend to concentrate on those ROM results that provide information about clinical symptoms and functioning. However, service user-oriented approaches promote a focus on personal recovery, which reflects the importance of finding meaning and giving value to personal experiences [7]. A second problem is that service users have limited access to ROM results and they are often not equipped to interpret them [8,9]. These problems need to be addressed, as research has shown that access to resources and the opportunity to share decision making has been indicated as a prerequisite for service users to become a more equal partner in communication with their clinicians [10,11]. Furthermore, shared decision making has been shown to improve the therapeutic alliance, and to lead to better care and treatment [12,13]. 

Since 2007, ROM assessments have been a regular element in care for people with psychotic disorders in the northern provinces of the Netherlands. The ROM protocol (called PHAMOUS), which is specifically developed for psychotic disorders, consists of a physical investigation (eg, weight, height, waist measurement, and glucose levels), multiple interviews and questionnaires concerning psychiatric and psychosocial issues, and service user satisfaction [14]. All service users with schizophrenia who receive care from any mental health care organization involved take part in ROM assessment at least once a year. After completion of the assessment, the parameters of the ROM assessment are uploaded into a central database by clinicians and research nurses via a link in the patient’s service user’s electronic file. Currently, the ROM-results are only reported to clinicians. Clinicians are supposed to discuss the results with their patients so that they can mutually decide whether the course of treatment needs readjustment [15]. However, a large percentage of service users do not receive adequate feedback concerning their ROM-results, as clinicians are not yet accustomed to discussing ROM results with service users [16].

In an attempt to improve ROM practice and to increase potential for service user empowerment, we developed a prototype of a web-based support system that provides service users diagnosed with schizophrenia with personalised advice, based on their ROM results. By means of this support system, the current problems with ROM practice may be partly tackled. The personalized advice provides users with accessible information about their ROM results, which may enable them to participate in shared decision making, and pave the way to better care. Prior research has shown that people with psychotic disorders can work with web-based and computer-based systems, despite the severity of their symptoms [eg, 17-21]. Findings are, however, inconsistent as to the amount of support service users need in working with computers (eg, [18] versus [21]).

In the present study, we extended the existing research by investigating the usability of a web-based support system for ROM. We examined whether our support system can make ROM-results more accessible to service users and provide them with more concrete information that they can use to discuss their personal goals with their clinician. The aim of this paper is to provide a brief overview of the web-based system and to report on its usability from the perspective of service users with schizophrenia.

## Methods

### Content and Technology of the Web-Based Support System Prototype

The prototype of the web-based support system is called WEGWEIS, which is a Dutch abbreviation that stands for web environment for empowerment and individual advice. The WEGWEIS support system offers users advice about various topics related to psychiatric treatment, rehabilitation, and personal recovery. This advice is based on the service user’s ROM assessment results, as conducted in the northern provinces of the Netherlands. The support system is a website, which can be accessed by entering a username and a password (see Multimedia Appendix 1). The system is to be used by service users at home or in a clinical setting (eg, a community hospital).

When building the prototype, we focussed on two important and widely used ROM measures, namely the clinician-rated Health of the Nation Outcome Scales [22], which measures health and social functioning, and the service user-rated Manchester Short Assessment of Quality of Life [23], which measures quality of life. Based on item scores of these measures and using innovative algorithms combined with ontological reasoning, the system identifies specific health care problems for each individual service user and provides relevant and tailored advice [24]. The algorithms are innovative because they break with conventional case-based reasoning approaches in that they decouple symptoms from outcomes, allowing the outcomes to be dynamic [24]. The content of the advice consists of information derived from evidence-based research (eg, the Dutch Multidisciplinary Guideline for Schizophrenia), clinical expertise, and service user experiences.

When, for example, the ROM results indicate that a service user is experiencing physical problems, the system offers advice indicating that physical problems can be a side effect of medication, referring to the Dutch Multidisciplinary guideline for schizophrenia. Furthermore, the advice suggests that side effects may be resolved by adjustment of the medication. Service users are also referred to their psychiatrist – by name – for more information (see Multimedia Appendix 2). When service users appear to experience problems with personal safety, they are provided information about and linked to the local patient counsellor. They also have the opportunity to read about experiences of other service users (see Multimedia Appendix 3). In another example, service users who are troubled by hearing voices are provided a video showing someone suffering from the same condition and offering information about treatment options (see Multimedia Appendix 4). More information about the advice can be found elsewhere [25]. The algorithm for advice selection, as well as a brief overview of system design and architecture are presented elsewhere [24].

The prototype is created with open source software, using the Ruby on Rails Web-framework (http://rubyonrails.org/). The website uses secure connections for all traffic. Service users can access their ROM-results by logging in with a username and password, which are sent to them by email. Failed log-in attempts are logged by the system. ROM-results can only be accessed via patient accounts.

### Development of the Prototype

The prototype of the web-based support system was developed by a multidisciplinary team of computer, social, and medical scientists in close collaboration with a group of service users with a schizophrenia spectrum disorder. The content and functionality of the first prototype was based on a needs assessment (unpublished material) conducted in 2009, consisting of semi-structured interviews with service users, relatives of service users, nurses, psychologists, psychiatrists, and people involved in e-mental health services for people with a psychiatric disability.

We put particular focus on the design of the support system’s user interface, as it has been suggested that people with schizophrenia have special needs with regard to web design [26]. This is supported by the theory that the quality of a user interface is partly determined by the extent to which users are able to create a so-called mental model of the website. A mental model can be described as a representation of a person’s thought processes regarding the functionality and structure of the website, and the flow of information therein. Therefore, it is important for designers to match as closely as possible the user interface with this mental model [27]. Finding a good match can be particularly challenging. This is especially the case when dealing with people with schizophrenia, who experience cognitive problems such as concentration, memory and information processing difficulties [26]. As a result, their mental models may differ from those of other users.

A few studies have investigated the challenges in web design for people with a schizophrenia diagnosis. Results from these studies suggest that users with schizophrenia experience difficulties with stimulus overflow, large amounts of text or information, interpretation of two-word labels, and remembering previous steps in the navigation process [17,18,26,28]. Furthermore, some of them experience paranoia when using computers and Internet [17].

In conjunction with the general guidelines as described in *User Interfaces for all* (a handbook for user interface design) [29] and taking into account the findings from prior research, we set out some specific rules for the design of the support system’s interface. The most important of these specific rules were the following: no use of unexpected pop-ups, transparency of procedures (ie, clear information about what happens when users click a button, what purposes their personal information is used for and who it is available to, etc), use of concrete descriptions (including using the name of a service user’s psychiatrist, instead of the general designation ‘your psychiatrist’), limited amount of text on one screen with an option to increase/decrease the amount of information, use of video material in addition to text, limited number of bright colours and avoiding jargon or difficult terms.

### Participants

Service users were recruited from four mental health care organizations in the Netherlands through snowball sampling. Snowball sampling involves asking a key informant or study participant whether they can suggest a person who fits the study criteria and asking them to introduce this person to the researcher [30]. In our case, study participants were recruited by 5 clinicians and fellow study participants. The study was conducted in March and April 2011. The inclusion criteria were (1) having a diagnosis of schizophrenia or a related psychotic disorder (eg, schizo-affective disorder, schizophreniform disorder, schizotypal disorder), (2) being between 18 and 65 years old and (3) being fluent in Dutch. There were no exclusion criteria.

Sixteen service users were asked to participate and a total of 15 service users, 10 male and 5 female, agreed to participate in the study. The age of the participating service users ranged from 23 to 61 years, with a mean age of 42. The duration of illness for 13 of these service users was known and ranged from 3 to 25 years, with a mean duration of 13 years. All service users received care in an outpatient setting except for one, who was committed in a forensic setting. In order to provide participants with some time to consider their participation, they were informed about the purpose and content of the testing by either a clinician or one of the experimenters (LvdK) at least a week prior to testing. Directly before the usability testing was to start, written informed consent was obtained. After completing the study, participants received a gift voucher of 15 euros.

Four Information and Communication Technology (ICT) experts participated in the study. They fulfilled the role of evaluator in a heuristic evaluation process, as described below. All ICT experts were experienced in developing ICT applications for mental health care organisations.

### Usability Testing

Usability can be defined as the ease with which users can use a particular tool or object to achieve a specific goal. One of the leading experts on usability, Jakob Nielsen, distinguishes five main quality components of usability [31]: (1) *learnability*: how easy is it for users to accomplish basic tasks the first time they encounter the design; (2) *efficiency*: once users have learned the design, how quickly can they perform tasks; (3) *memorability*: when users return to the design after a period of not using it, how easily can they re-establish proficiency; (4) *errors*: how many errors do users make, how severe are these errors, and how easily can they recover from the errors; and (5) *satisfaction*: how pleasant is it to use the design.

Usability can be assessed by usability testing. There are three testing categories: heuristic evaluation, qualitative evaluation, and quantitative evaluation. These categories will be described in the following sections.

#### Heuristic Evaluation

We started the usability testing by conducting a heuristic evaluation. This is a research method for detecting usability problems with the interface early in the testing process [31]. Heuristic evaluation is conducted by evaluators and takes place prior to the testing by end-users (in our case service users). Problems detected by the evaluators are dealt with immediately so they do not influence the rest of the testing process.

Heuristic evaluation is usually conducted by more than one evaluator because it is difficult for one person to detect all usability problems. We appointed four ICT experts to fulfill the role of the evaluator, as this falls into the range of the optimal number [32]. The process of heuristic evaluation used in this study is based on Nielsen’s recommendations [33]. The evaluators were given a brief introduction to the background and rationale of the web application under review, then given instructions on how to conduct the heuristic evaluation. One of the most important instructions was that they were not allowed to communicate with each other during the testing process. Then, the evaluators sat at the computer and went through the user interface according to a scenario written by the experimenters. The scenario included using log-in procedures, username and password retrieval processes, font size modification, completing questions, going through advice units, printing information, searching for advice by means of key words, and providing feedback about the website. The evaluators inspected the interface independently, assessing the various elements based on a list of ten recognized usability principles (“heuristics”) [33] translated into a series of questions (see Table 1). Their findings were put in a template developed by the experimenters.

The data in the four completed templates was assembled in one document and its content was analysed, meaning that the data was categorized according to Nielsen’s usability topics [33] (see also Table 1). Finally, a list of usability violations was created and sorted according to frequency and priority. A debriefing meeting was organized with evaluators and the experiments, during which the results of the heuristic evaluation were discussed during a brainstorm session in a brainstorm mode. Decisions were made as to which usability issues were considered most urgent and how these issues could best be solved.

**Table 1:** Assessment criteria for heuristic evaluation

Usability principle	Question
1. Visibility of system status	Are there any incidents where the website is unresponsive or slow?
2. Match between system and the real world	Are there any words/sentences used on the website that do not match the language used by the intended group of users?
3. User control and freedom	Are there any instances where important changes made by users cannot be easily undone?
4. Consistency and standards	Are there any inconsistencies concerning language use or functionality?
5. Error prevention	Are there any instances where users can easily make mistakes? Before executing an action, are users asked for confirmation where needed?
6. Recognition rather than recall	Are there any pages where the content or structure is unclear or insufficiently explained?
7. Flexibility and efficiency of use	Are there any frequently used functionalities on the website that are not accessible fast enough?
8. Aesthetic and minimalist design	Are there any instances in which the website offers too much information, whereby the user can loose track of the situation?
9. Help users recognize, diagnose, and recover from errors	Are there any error alerts which are not clear to users, which do not identify the problem correctly or do not provide a solution?
10. Help and documentation	Is there enough help or documentation available?

#### Qualitative Evaluation

After completion of the heuristic evaluation, we conducted a qualitative evaluation. In this process, end-users fulfilled the role of the evaluator. The participants were invited to sit at a computer. We then asked them to use the web application following a scenario written by the experimenters (the same scenario as used in the heuristic evaluation). Users were encouraged to work through the scenario step by step, starting with the log-in procedures. We decided not to ask participants to think aloud, as we suspected that this might affect their way of working substantially. Two-thirds of the end-user participants carried out the testing at our research centre. During the testing, one of the experimenters observed the users' actions via a beamer projection on a screen, while making notes (see Multimedia Appendix 5). One-third of the users conducted the testing at home on their own computer and were joined by an experimenter who observed from a distance. When users finished the testing, they were asked to verbally describe their first impression of the support system. As the main aim of this part of the testing was to find out how users interact with the web system, the research method used in this qualitative evaluation was (non-participant) observation [34]. One experimenter (LvdK) was present during the testing session and made notes (using paper and pencil) which indicated how participants worked their way through the scenario. The sessions were not audiotaped, as observation was the main evaluation method and we assumed that participants might not feel at ease with audiotaping. The verbal information provided by service users was analysed by identifying positive and negative feedback items.

#### Quantitative Evaluation

After the qualitative evaluation was completed, a quantitative evaluation was conducted. End-user participants were asked to fill out a short questionnaire, consisting of 5 questions measured on a 5-point Likert scale. They were asked about their computer and Internet use. This questionnaire was derived from another European study testing a web application developed for a comparable group of end-users [18]. Furthermore, participants completed a Satisfaction Questionnaire, measuring their satisfaction with various aspects of the web application concerning layout, structure, user-friendliness and content. This questionnaire consisted of 13 statements to be subsequently rated on a 7-point Likert scale, ranging from completely disagree (1) to completely agree (7). The Satisfaction Questionnaire was specifically designed for this study by the research group. Descriptive analysis (mean, standard deviation) of the quantitative data was conducted with SPSS 16.0 statistical software for Windows (SPSS Inc., Chicago, IL, USA).

## Results

The results of the usability tests are a combination of the three categories of testing mentioned above, namely heuristic evaluation, qualitative evaluation, and quantitative evaluation.

### Heuristic Evaluation

All ICT experts evaluating the website were able to complete the scenario written by the experimenters. No major problems were reported with regards to language, undoing changes, structure or content of the pages, accessibility of functionality and clarity of error messages (ie, usability principles 2, 3, 4, 6, 7 and 9). However, there were some instances in which the website was unresponsive or slow. Furthermore, at times the website seemed to offer too much information at once, and three situations occurred whereby users were not clearly directed to the right page. The most obvious problem reported was that the Disclaimer page was empty and that there was no existing Help section or Frequently Asked Questions section.

During the debriefing meeting, all problems were discussed and decisions were made on how to solve problems most effectively. All problems were solved prior to the qualitative and quantitative testing with service users, except for the missing Frequently Asked Questions section, which was composed after the usability testing with service users. 

### Qualitative Evaluation

All end-user participants were able to complete the scenario, although three of them needed some hints in order to continue to the next step. For instance, one participant had difficulty finding out how to adjust his personal profile, and the experimenter had to explain how he could access the profile. Although the participants were not asked to think aloud during the evaluation, most of them did so spontaneously. One of the difficulties expressed was that some buttons were hard to find or that their function was not entirely clear. One example is the ‘Feedback’ button. This button was located at the left part of the webpage, situated vertically and separately from the navigation bar. Three participants could not immediately locate it and two did not know what to use it for. Furthermore, several participants suggested that the website could be made more attractive by using more colour, more images and videos, and more links. However, others indicated they were happy with the layout and found the website to be nice and simple.

With reference to the content of the website, participants expressed that they recognized many issues that people suffering from schizophrenia are faced with and believed that the website could be a useful instrument in supporting people in their personal recovery process. In addition, while reading the advice, various service users came up with relevant information that they thought should be added to the advice. A few other participants, however, stated that the information about illness symptoms and medication should be more extensive. In addition, one participant suggested creating a possibility for online communication between clinicians and service users within the system.

### Quantitative Evaluation

The participating end-users reported to be well experienced in using computers and the Internet, to have good computer and Internet skills (see Table 2) and to have a positive attitude towards technology (see Table 2). There was one participant who reported to have almost never used the Internet. He appeared not to have access to the Internet, due to the fact that he was a forensic service user admitted into a penitentiary where Internet use was not allowed.

The mean score of satisfaction with the web-based support system prototype was 73.60 (the maximum being 90) with a standard deviation of 6.64. Ratings of the individual statements are presented in Table 3. As this table shows, the most disagreement amongst the participants concerned the question of whether or not the website was boring. This is in line with the results of the qualitative analysis, which showed that some participants found the website nice and quite simple, whereas others suggested that it could be improved by using more colour, images, and so on.

**Table 2:** Service users’ computer and Internet use, skills and attitudes

	Questionnaire response option	No. (n=15)
Computer use	Almost never	0
	Less than once a month	0
	Monthly	0
	Every week	1
	Every day	14
Internet Use	Almost never	1
	Less than once a month	0
	Monthly	0
	Every week	1
	Every day	13
Computer Skills	Very bad	1
	Bad	0
	Not bad, not good	5
	Good	8
	Very good	1
Internet Skills	Very bad	1
	Bad	0
	Not bad, not good	4
	Good	9
	Very good	1
Attitude towards computers	Very negative	0
	Negative	0
	Neutral	0
	Positive	11
	Very positive	4

**Table 3:** Results of Satisfaction Questionnaire

	Mean (SD)	Percentage (and absolute number) of service users who agreed (score 6) or completely agreed (score 7) with the statement (N = 15)
I can easily find my way on the website	5.73 (0.88)	80 (12)
I am satisfied with the language used on the website	6.13 (0.35)	100 (15)
The website is boring	3.13 (1.55)	7 (1)
I am satisfied with the font used on the website	5.87 (0.83)	93 (14)
The colour of the website was appealing	5.33 (1.35)	67 (10)
The website does not contain distracting elements	5.8 (1.21)	80 (12)
The advice provides me with meaningful information	5.67 (0.72)	80 (12)
The amount of information in the advice is too much	2.87 (1.55)	7 (1)
The advice can help me reflect on what I want	5.73 (1.16)	80 (12)
I can imagine myself discussing the advice with my clinician in the future	5.67 (1.11)	80 (12)
I can imagine the advice being helpful to others	6.27 (0.46)	100 (15)
I think I will use the website in the future	5.53 (0.83)	60 (9)
I would recommend the website to others	5.87 (0.64)	86 (13)

## Discussion

In this study, we investigated the usability of the first prototype of a web-based support system for people diagnosed with schizophrenia. The heuristic evaluation with ICT experts revealed some minor problems; the most important ones of which were unresponsive or slow processing of information, too much information displayed at once, an empty Disclaimer page and no existing Help section. The first three problems were solved before testing with service users. During qualitative testing, our group of end-users reported some difficulties with, among other things, the location and function of the ‘Feedback’ button and with understanding how to adjust one’s personal profile. In addition, several suggestions were made to make the interface more attractive. These results indicate that the end-users involved in this study, varying in age, sex and duration of illness, were able to use the support system easily. Furthermore, the content of the advice generated by the support system was judged to be meaningful and supportive. We can therefore conclude that, overall, the support prototype has valuable potential for improving the ROM practice and that it is worthwhile to develop it further into a more mature system.

### Comparison With Existing Research

Our preliminary results are in line with previous research, which shows that people with psychotic disorders can work with web-based and computer-based systems [17-21], but there are some differences between our research and that of others that we need to address.

Whilst designing the interface, we followed some specific rules based on existing literature in the field and for this group of end-users as well as applying general rules of interface design. However, we did not comply with all recommendations presented in the literature as feedback from individual service users during the design process, which took place prior to the usability testing (not described in this paper), suggested it might not be necessary. For instance, we decided to use a bright background colour (yellow) for the web pages, and we used arrow heads and drop down menus instead of pop-ups, which was advised against by Rotondi et al, [20]. However, these deviations did not result in any usability violations.

This may be explained by the fact that there appears to be a difference between basic principles for user interface design and concrete applications thereof. Each basic principle can be translated into various concrete applications. If the principle is to avoid an abundance of information, this can be achieved by either limiting the amount of text on one page, or by ordering the information in a surveyable way. Both forms can be effective, depending on, among other things, users’ individual preferences. Furthermore, as the functionality of Internet browsers develops very quickly and new innovations emerge, some earlier problems with the user interface may be no longer relevant. For instance, Rotondi et al [20] discourage the use of an absolute font size that cannot be enlarged. Given the flexibility of modern-day browsers, however, this is hardly an issue anymore, as font sizes can be adjusted rather easily.

Another issue to be addressed is the context for which the support system is developed. As mentioned before, our system is intended for independent use by service users at their home or on a hospital ward. This is in line with the study by Bickmore et al [21], who developed a computer-based medication adherence system with relational agents for service users with schizophrenia, to be used at home and without assistance or interpretation from clinicians. Results of their pilot evaluation study (*N =* 16) showed that independent use of the computer system was acceptable for all but one of the study participants, who were recruited at an outpatient clinic. However, these results seem to contradict with the findings of Kuosmanen et al [18], who reported that service users with psychotic symptoms needed support from nurses in using their web system. This difference in findings could be explained by symptom severity of service users, as the study by Kuosmanen et al [18] was conducted in a locked-door setting, while the one by Bickmore et al [21] and our study primarily involved service users staying at home.

The results of our study add to previous studies in that usability tests suggest that there need not be insurmountable barriers in independent use of web-based systems for people with psychotic disorders. However, we need to investigate the system in a real world setting in order to draw broader conclusions. In future research, the most important question will be not so much whether or not service users with psychotic symptoms can independently work with web systems, but rather, under what conditions they can successfully work with them. These conditions may depend upon the service users’ circumstances, such as receiving care in an inpatient or outpatient setting, severity of specific symptoms (eg, paranoid ideas), and, of course, the level of computer experience. In addition, they might also be related to the web-system, such as the content and the complexity of the system's functionality.

### Future Development of Our Web-Based Support System

In future development of our web-based support system, several issues need to be taken into account. First, in order to provide end-users with a support system tailored to their needs and preferences, a flexible interface is needed. Some users like a colourful background and all kinds of multimedia elements, whereas others prefer a more simple interface. This calls for an interface which can be customised. Furthermore, in order to keep the content of the advice oriented toward the service user and to work in a more Health 2.0/Medicine 2.0 fashion [35], we need to facilitate users in adding information to the advice by creating options to post comments or upload material. In addition, possibilities should be explored for interactive communication among the service users themselves and between the service users and clinicians.

With regard to the support system’s technology, we aim to develop more sophisticated advice algorithms and enlarge our data set so that the advice offered to service users can be even more personalised. Furthermore, we will explore interoperability and connectivity with personal health records and electronic patient files, and integration with successful platforms currently used in mental health care.

### Limitations

Our study should be viewed with consideration of certain limitations that we encountered. First, our sample of service users was small and we used a method of snowball sampling, which is a form of convenience sampling. One disadvantage of convenience sampling is that one runs the risk of compiling a non-representative study sample. In our case, the study sample was quite diverse in age, sex, and duration of illness, which favours the sample’s representativeness.

In contrast, what appears to be less favourable for the sample’s representativeness is the fact that the service users recruited for this study might have had a particular interest in working with computers and websites, which could have affected our results. This could be the case given that the service users concerned were reported to be quite skilled in using the computer and Internet. However, we need to take into account that the Netherlands is one of the countries with the highest Internet penetration rates. In March 2011, 88.3% of the Dutch population had Internet access, while the world wide average is only 30.2% [36]. This suggests skillful computer and Internet use is not uncommon in the Netherlands. Understandably, there will be differences between the level of computer and Internet skills of the general Dutch population and people with mental disorders. However, we believe that the representativeness of our sample on this point does not necessarily invalidate our conclusions.

Second, the presence of an experimenter during the testing session may have affected the behaviour of service users conducting the testing. Although the experimenter encouraged participants to mention both strong and weak features of the web application, they might have felt reluctant to be critical.

Third, the support system was not tested in the context of a full ROM assessment, but as a somewhat isolated part thereof. Therefore, at the moment, we cannot gain a comprehensive view of the system’s functioning in its full setting. This issue needs to be addressed in future research in a clinical evaluation, followed by an examination of its effectiveness in a randomized controlled trial, in order to determine whether or not the present system can genuinely contribute to improving ROM practice.

## Multimedia Appendix 1

Homepage Wegweis website.

## Multimedia Appendix 2

Advice can refer to the Dutch Multidisciplinary Guideline and encourage service users to contact their psychiatrist.

## Multimedia Appendix 3

Users have the opportunity to learn about the experiences of other service users.

## Multimedia Appendix 4

Service users can watch a video of someone who experiences hearing voices.

## Multimedia Appendix 5

Test situation at our research center. During the testing, one of the experimenters observed the users' actions via a projection on a screen while making notes.

## Abbreviations

ICT: Information and Communication Technology
ROM: Routine Outcome Monitoring
WEGWEIS: name of the web-based support system described in this study. WEGWEIS is a Dutch abbreviation that stands for web environment for empowerment and individual advice.
