# Supplementary material for: Usability Evaluation of a Web-Based Support System for People With a Schizophrenia Diagnosis
Source: J Med Internet Res. 2012 Feb 6;14(1):e24. doi: 10.2196/jmir.1921 (PMC3374538; doi:10.2196/jmir.1921)
Supplement: Supplementary file 1 [file jmir_v14i1e24_app1.pdf]

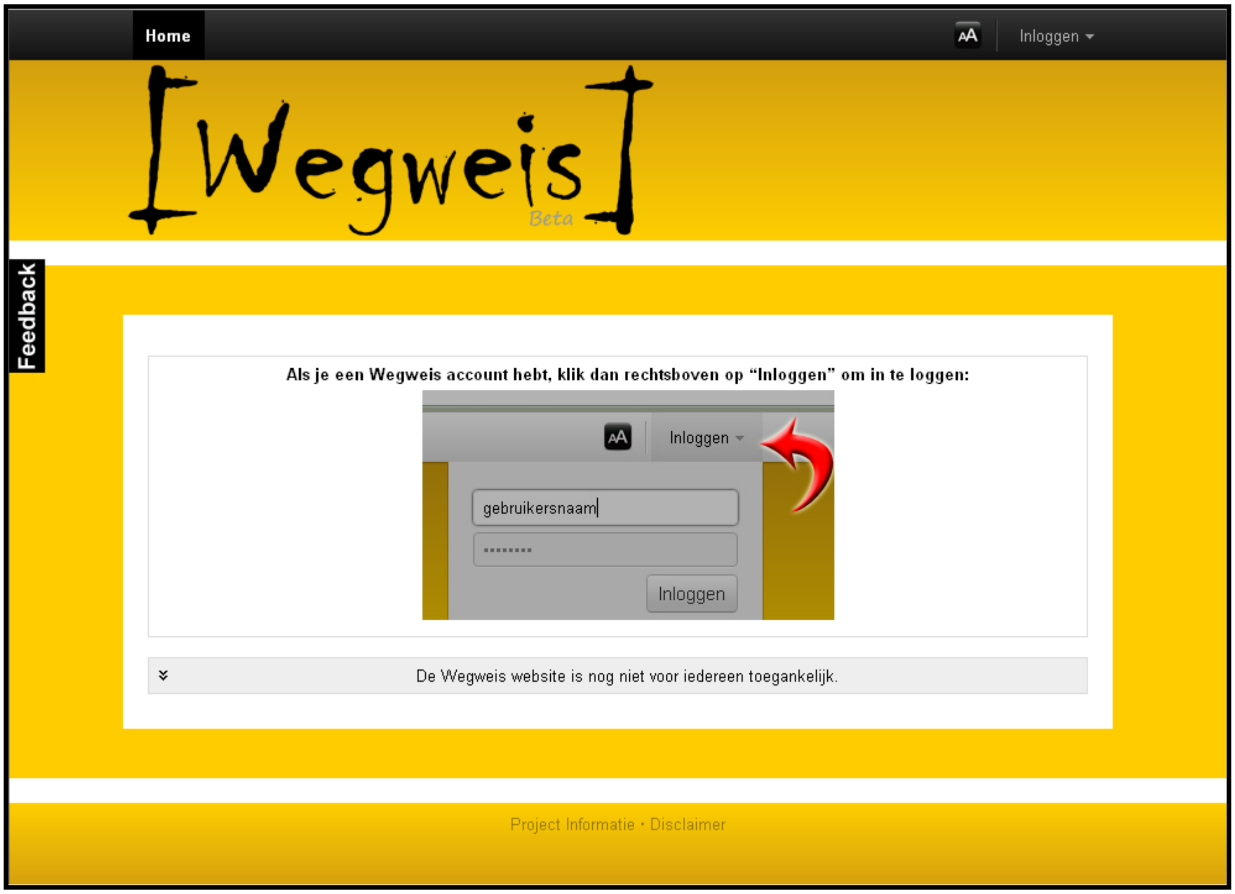

Translation:
If you have a Wegweis account, click on "Inloggen," in the upper right corner, to log in.
The Wegweis website is not publicly accessible.

You can find more information on: development.wegweis.nl or at our weblog: blog.wegweis.nl
