# Supplementary material for: Usability Evaluation of a Web-Based Support System for People With a Schizophrenia Diagnosis
Source: J Med Internet Res. 2012 Feb 6;14(1):e24. doi: 10.2196/jmir.1921 (PMC3374538; doi:10.2196/jmir.1921)
Supplement: Supplementary file 2 [file jmir_v14i1e24_app2.pdf]

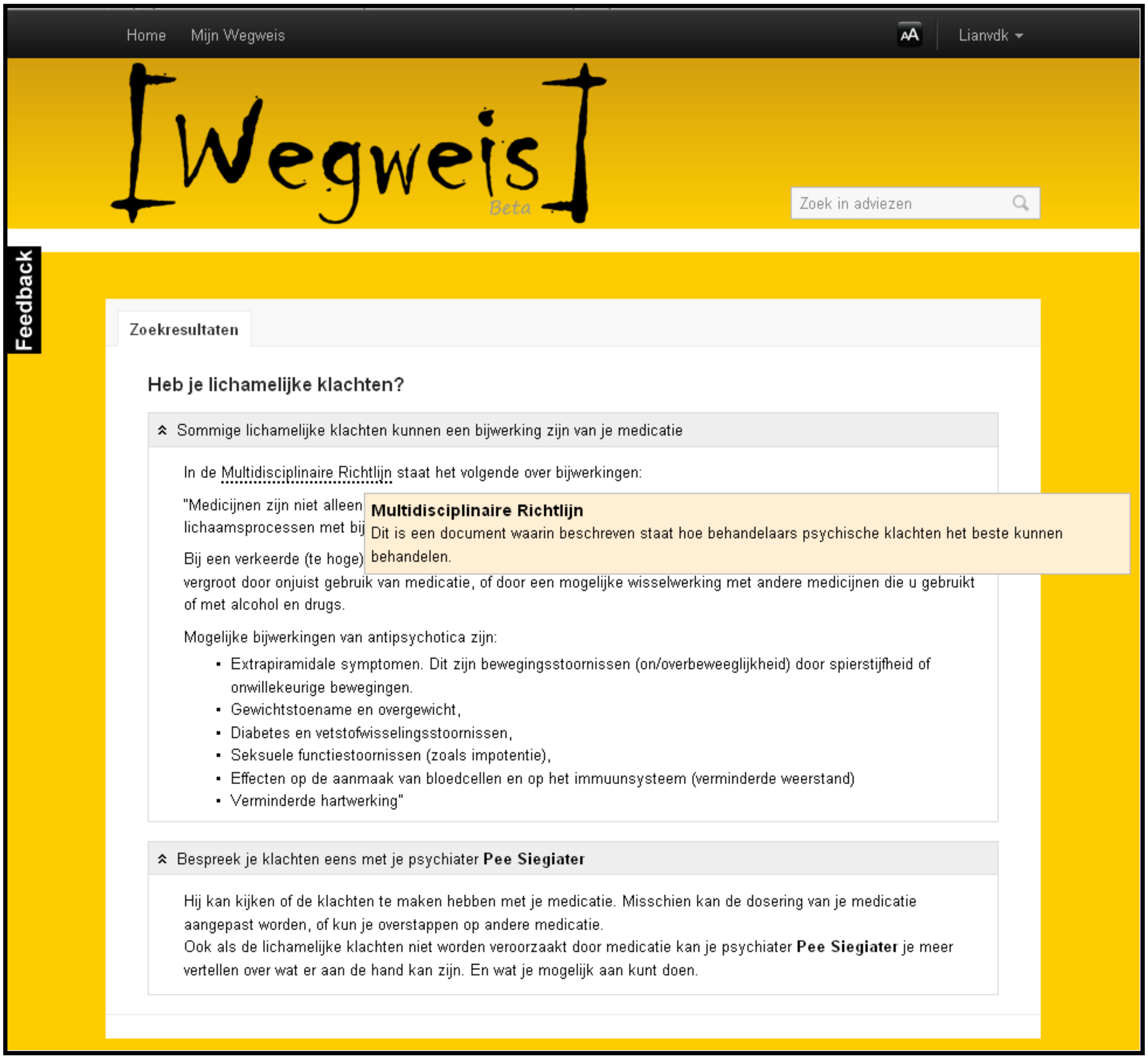

Translation:
Do you have any physical complaints?
Some physical problems can be a side effect of your medication.
The multidisciplinary guideline [Multidisciplinary Guideline: this is a document which describes how clinicians should treat psychiatric problems] states the following:
“Medication does not only affect those parts of the body where an effect is desired. As a side effect, they also affect other body processes. A wrong or too high dosage increases the risk of side effects. The risk of side effects also increases when medication is not used properly, when medications are combined, or when used in combination with alcohol and drugs. Possible side effects of antipsychotics are:

- Extra pyramidal symptoms. These are movement disorders characterized by stiffness of the muscles or involuntary movements.
- Weight gain and obesity
- Diabetes and metabolic syndromes
- Sexual dysfunctions
- Effects on the production of blood cells and on the immune system (decreased resistance)
- Decreased heart function”

Feel free to discuss your symptoms with your psychiatrist Pee Siegiater
He can tell you whether your physical symptoms are related to the use of your medication. Perhaps the dosage of your medication can be adjusted, or you can change medication. If your symptoms are caused by something other than your medication, your psychiatrist Pee Siegiater can also tell you more about possible causes and what you can do about it.
