# Supplementary material for: Usability Evaluation of a Web-Based Support System for People With a Schizophrenia Diagnosis
Source: J Med Internet Res. 2012 Feb 6;14(1):e24. doi: 10.2196/jmir.1921 (PMC3374538; doi:10.2196/jmir.1921)
Supplement: Supplementary file 3 [file jmir_v14i1e24_app3.pdf]

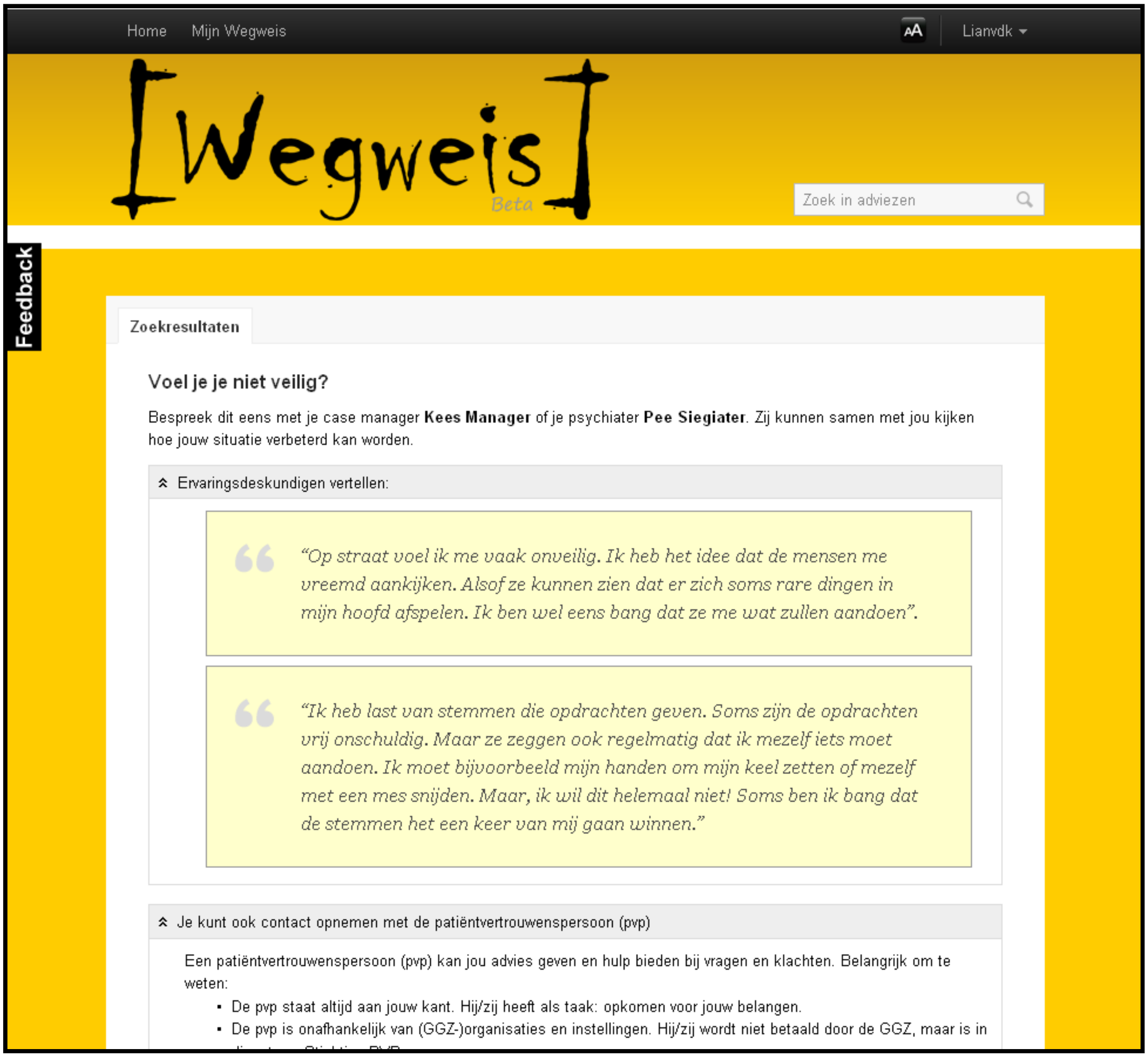

Translation:

Do you feel unsafe?
You can discuss this with your case manager Kees Manager or your psychiatrist Pee Siegiater. Together, you can discuss how your situation can be improved.

Experiential experts:
“I often feel unsafe when out-and-about. I have the feeling that people look at me strangely. As if they can see that sometimes strange things are happening in my head. Sometimes, I am afraid that they will hurt me.”
“I suffer from voices that give me orders. Sometimes these orders are fairly innocent. But they also regularly tell me to harm myself. For instance, they tell me to put my hands around my throat or to cut myself with a knife. But I don’t want to do that at all! Sometimes, I am afraid that the voices will win”.
You can also contact your patient counsellor
A patient counsellor can give you advice and support when you have questions or a complaints. It is important to know that:
- The patient counsellor is always on your side. It is his/her job to protect your interests.
- The patient counsellor is independent of the mental health care organisations. He/she is not paid by the mental health care organisations.
