# Supplementary material for: Usability Evaluation of a Web-Based Support System for People With a Schizophrenia Diagnosis
Source: J Med Internet Res. 2012 Feb 6;14(1):e24. doi: 10.2196/jmir.1921 (PMC3374538; doi:10.2196/jmir.1921)
Supplement: Supplementary file 4 [file jmir_v14i1e24_app4.pdf]

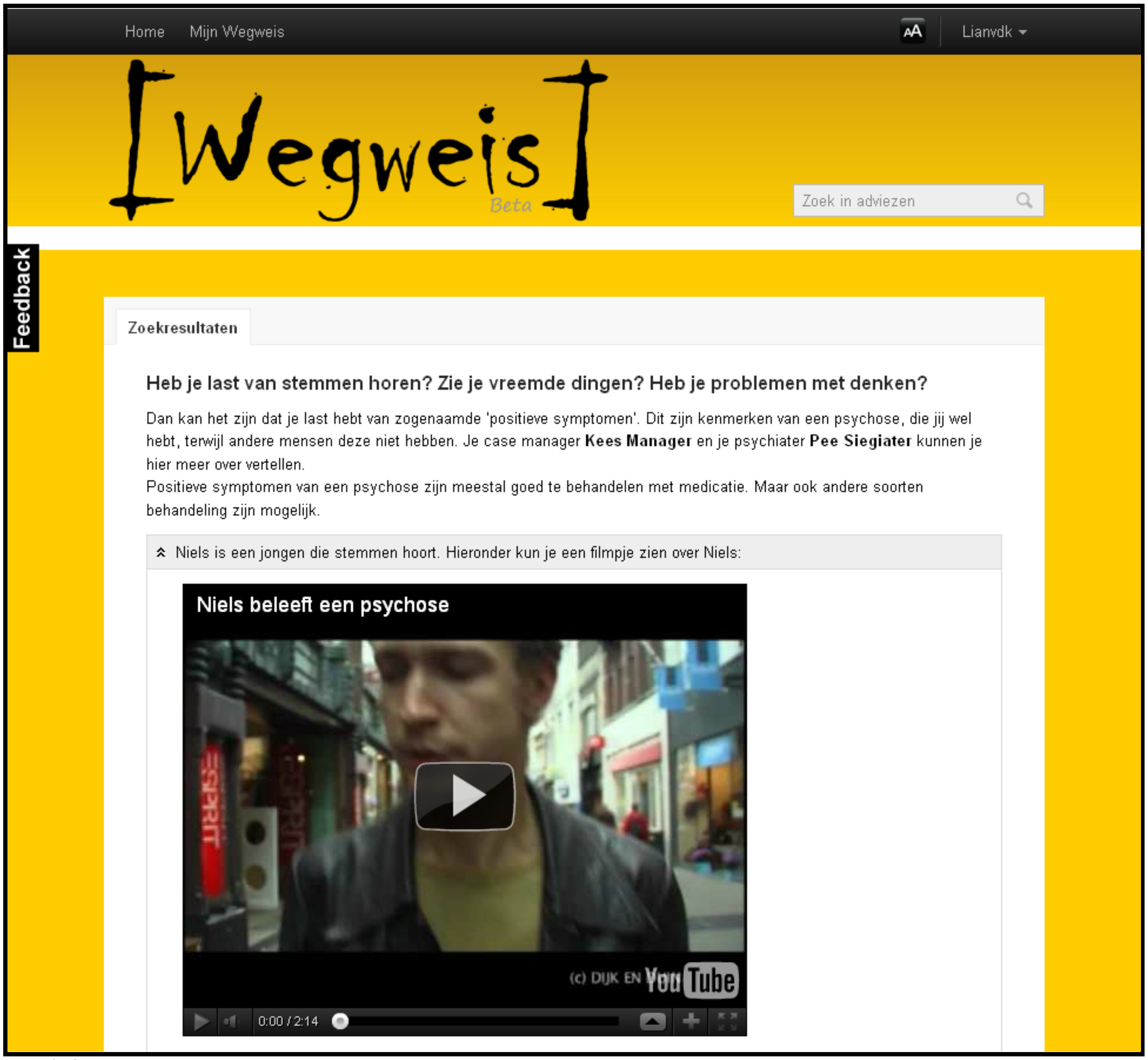

Translation:
Do you suffer from hearing voices? Do you see strange things? Do you suffer from having strange thoughts?

You may be experiencing so-called 'positive symptoms'. These are characteristics of a psychosis that you do have, but other people don't. Your case manager Kees Manager and your psychiatrist Pee Siegiater can tell you more about this.
Positive symptoms of a psychosis can often be treated with medication. There are also other forms of treatment available.

Niels is a boy who hears voices. Below, you can watch a short film about Niels.
