# Supplementary figures and images for: Usability Evaluation of a Web-Based Support System for People With a Schizophrenia Diagnosis
Source: J Med Internet Res. 2012 Feb 6;14(1):e24. doi: 10.2196/jmir.1921 (PMC3374538; doi:10.2196/jmir.1921)

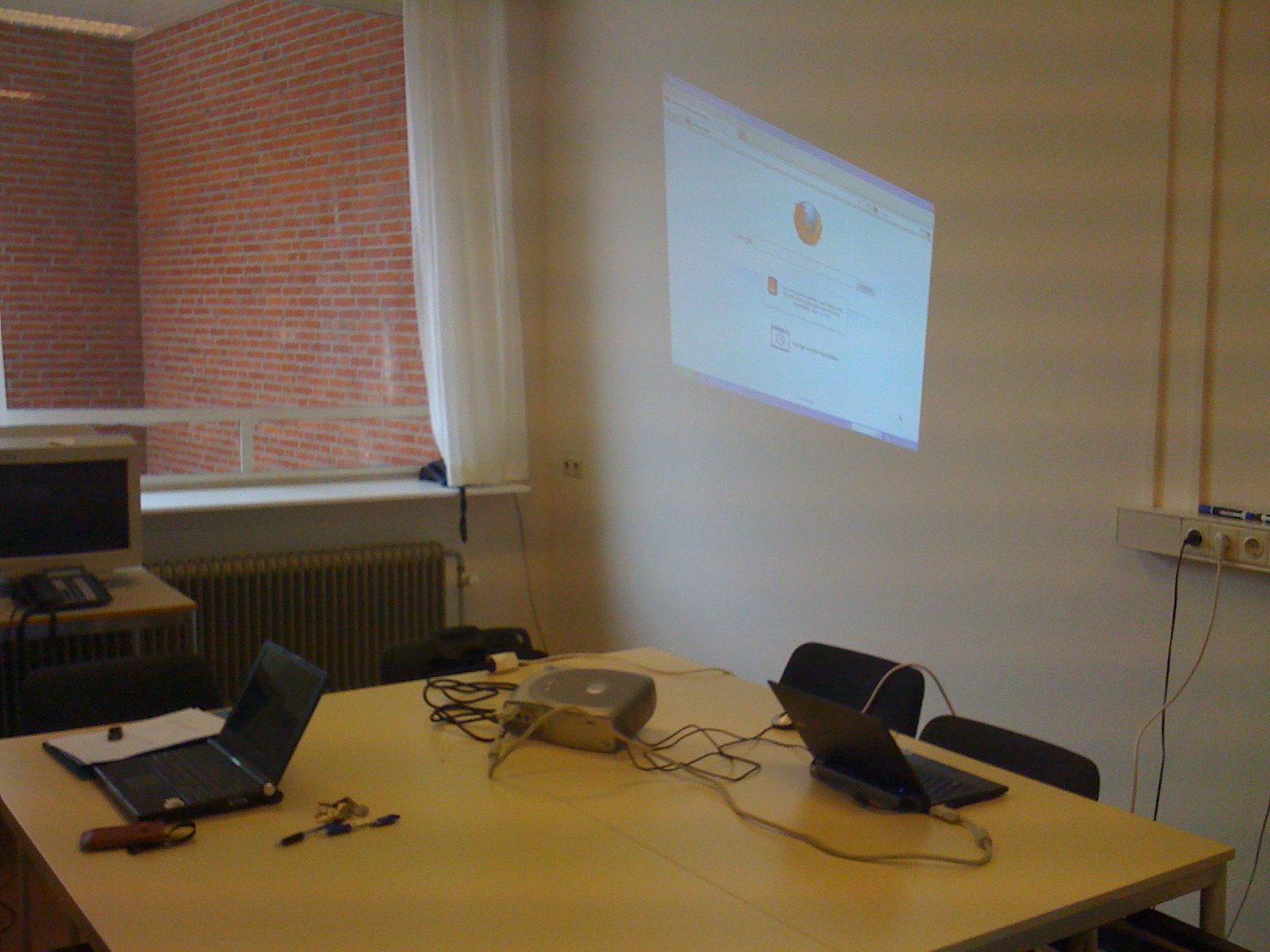

Supplement: Supplementary file 5 [file jmir_v14i1e24_app5.JPG]
